# Case Review of Sarcoidosis Resembling Sjogren's Syndrome

**DOI:** 10.4021/jocmr482w

**Published:** 2010-12-11

**Authors:** Yuhanisa Ahmad, Nur Shuhaila Shahril, Heselynn Hussein, Mohd Shahrir Mohamed Said

**Affiliations:** a Medical Department Hospital Putrajaya, Malaysia; b Medical Department UKM Medical Centre, Malaysia; c Medical Department Universiti Sains Islam Malaysia

## Abstract

We would like to report a case of a 29-year-old male patient who presented with multiple lymphadenopathy and vague symptoms of low grade fever, cough, weight loss, rashes, vomiting, dry eyes and dry mouth. Physical examination revealed submandibular lymphadenopathy, vasculitic rashes over both lower limbs, and parotid gland enlargement. Blood investigations showed mild anemia with leukocytosis, predominantly eosinophilia and high erythrocyte sedimentation rate and C-reactive protein. Computed tomography of the neck, thorax and abdomen showed bilateral submandibular, submental adenopathy, mediastinal and para-aortic lymphadenopathy with generalized reticulonodular densities in both lower lobes. There were hepatomegaly and bilateral enlarged kidneys with renal cyst. Histopathological examination from the cervical lymph node later revealed non-caseating granuloma, consistent of sarcoidosis. Patient responded well to prednisolone 50 mg daily with subsequent reduction in the size of cervical lymphadenopathy and parotid swelling.

**Keywords:**

Lymphadenopathy; Granuloma; Sjogren; Sarcoidosis

## Case Report

**Figure 1.**
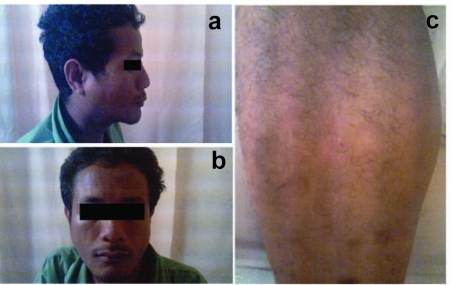
Right parotid swelling noted from lateral view; (b) Right parotid swelling; (c) Vasculitic rash over right lower limb.

A 29-year-old Malay male was referred to the rheumatology team for suspicion of Sjogren's syndrome. He presented with symptoms of malaise, low grade fever, occasional night sweats and dry cough for three months. It was associated with neck swelling for 2 months, which was progressively increasing in size. He also complained of pain during swallowing for one month and vomiting after half an hour taking meal. He claimed that he had been losing weight, but could not quantify it. He felt his eyes and mouth were dry for the past 2 months, which he needed to drink water frequently. He also noticed rashes over the upper limbs and lower limbs, which initially was red and itchy and later became papules and healed with hyperpigmentation (Fig. 1). There were no photosensitivity, malar rashes, oral ulcers or alopecia. He denied of any history of high risk behavior.

Physical examination revealed a well looking, thin man, not tachypnoeic with good hydration status. Blood pressure was 120/75 mmHg, pulse rate was 80 beats per minute and he was afebrile. Cardiovascular, respiratory and abdominal examinations were normal. Both parotid glands were enlarged and there were multiple cervical lymphadenopathies ranging from 0.5 to 3 cm in size, matted over the submandibular region which was not tender. There were also multiple vasculitic lesions over both lower limbs with hyperpigmented skin lesion. However, there were no malar, oral ulcers or photosensitivity rashes noted.

Initial investigation done in a private medical centre showed lowish hemoglobin with borderline high total white cell count: hemoglobin 13.4 g/dl, total white cell count 10.7 x 10^9^/L (normal 4 - 10 x 10^9^/L) and platelet count of 415 x 10^9^/L (normal 150 - 450 x 10^9^/L). Antinuclear antibody was negative, rheumatoid factor was positive (16 IU/ml) and tuberculosis screening was negative. Computed tomography (CT) of the neck showed bilateral submandibular adenitis with bilateral submandibular and submental adenopathy. Fine needle aspiration and cytology of the right submandibular swelling showed polymorphic populations of lymphoid cells. There was no granuloma or malignant cells seen. Ziehl-Neelsen stain for acid fast bacilli was negative.

Initial impression was systemic vasculitis and patient was admitted to ward for further workup. Repeated hemoglobin level was 13.6 g/L, total white cell count 12.1 x 10^9^/L, neutrophil 75.7%, lymphocyte 6.32%, eosinophil 14.9% and platelet count 368 x 10^9^/L. Full blood picture showed mild eosinophilia with inflammatory features. Inflammatory markers were high whereby erythrocyte sedimentation rate (ESR) was 74 mm/hr while C-reactive protein was 36.1 mg/L. There was renal impairment noted as the serum creatinine ranged from 230 to 275 umol/L. Urea level was between 7 - 10.3 mmol/L. Serum sodium, potassium, calcium and phosphate were within normal limits. Liver enzymes were normal except for serum alkaline phosphatase which was increased up to 329 U/L. Total protein was high, predominant in globulin ranging between 60 - 66 g/L. Hepatitis screening was negative. Repeated antinuclear antibody, anti Rho, anti nuclear cytoplasmic antigen (ANCA) and anti-mitochondrial antibody were negative. Anti-smooth muscle antibody was positive, titre 1:20. Serum immunoglobulin E was high, 741 ku/L (normal range < 100 ku/L). There was hypocomplementemia which serum C3 was 0.44 g/L and C4 was 0.05 g/L. Cytomegalovirus and Epstein Barr virus IgM were negative.

**Figure 2.**
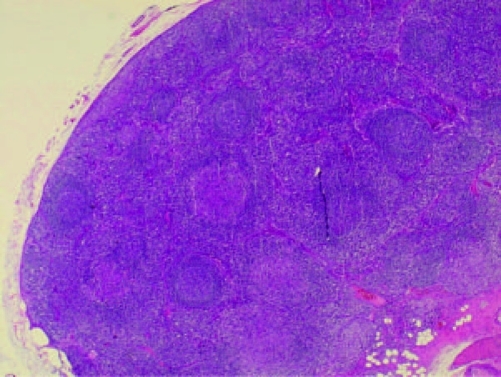
Cervical lymphnode biopsy showing multiple non-caseating granulomas.

**Figure 3.**
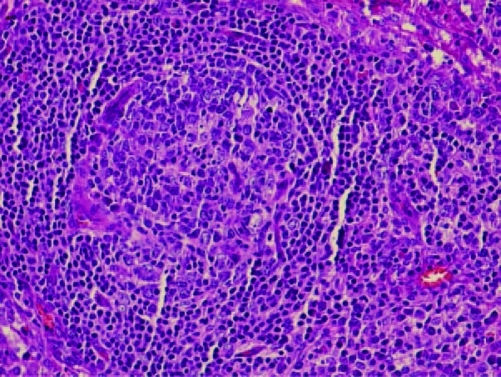
High power field non-caseating granuloma with surrounding lymphocytes.

Computed tomography of thorax and abdomen showed multiple nodes in both axillary (largest 0.9 cm in left axilla), mediastinal lymphadenopathy, paratracheal 1.1 cm, preaortic 0.8 cm, subcarina 1.8 cm, multiple subcentimeter para-aortic and aortocaval nodes (largest in para-aortic region measuring 0.7 cm) and generalized reticulonodular densities mainly in both lower lobes. The patient also had bilateral small inguinal lymph nodes (largest 0.8 cm in the left inguinal area). There were bilateral enlarged kidneys, left measuring 15.6 cm and right 13.4 cm with renal cysts. There was hepatomegaly, measuring 22.4 cm, homogenous with no focal lesion. Skin biopsy from the left leg showed features suggestive of resolving vasculitis. Lower lip tissue biopsy showed mild chronic inflammation. Excision biopsy from the left cervical lymphnode showed non-caseating chronic granulomatous lymphadenitis, consistent with sarcoidosis (Fig. 2, 3).

Tablet prednisolone 50 mg daily for 6 weeks was instituted for this patient, with tapering doses slowly over 6 months. He was discharged well with other medications: oral Omeprazole 20 mg bd, oral calcium carbonate 1g bd and oral calcitriol 0.25 mcg bd. Serum angiotensin converting enzyme levels came back later as 49 U/L which was normal.

Ophthalmology assessment showed normal visual acuity both eyes, no features of Sjogren's syndrome or uveitis.

During clinic follow-up, patient generally felt much better with reduction in size of cervical lymphadenopathy and parotid swelling. Oesophagoduodenoscopy (OGDS) revealed single ulcer at antrum and polyp at prepyloric region. He responded to oral Rabeprazole 20 mg daily. Gastric biopsy result showed hyperplastic polyp. He was lost to follow-up.

## Discussion

This case presented a diagnostic dilemma in the beginning in which this patient presented with cervical lymphadenopathy and vague symptoms of low grade fever, weight loss, rashes, dry eyes and dry mouth. The presentation that favors sarcoidosis were mediastinal lymphadenopathy with reticulonodular densities over the lung field, parotid swelling, and vasculitic lesion over both lower limb and high inflammatory markers. However, blood investigation did not show hypercalcemia or high level of angiotensin-converting enzyme level that occurred typically in sarcoidosis. The final diagnosis of sarcoidosis was based on the histopathological excision biopsy of the cervical lymphadenopathy.

Sarcoidosis is a multisystem granulomatous disorder characterized pathologically by the presence of non-caseating granulomas in involved organs, occurring in patients between 10 and 40 years of age in 70 to 90 percent of cases. It typically presents with bilateral hilar adenopathy, pulmonary reticular opacities, and skin, joint or eye lesions. Lung is the most common organ involved, which is 95% followed by skin and lymph nodes, 15.9% and 15.2% respectively [1, 2]

The incidence of the disease varies among geographical regions. It occurs more commonly, three to four times in blacks in the United States of America [3]. There are numerous reports of familial clustering of sarcoidosis. Several alleles confer susceptibility to disease, for example, HLA DR 11, 12, 14, 15, 17 while HLA DRI, DR4, and possibly HLA DQ*0202 seem to be protective [4].

The stage of pulmonary involvement is based on the chest radiograph. Stage I is defined by the presence of bilateral hilar adenopathy, often accompanied by right paratracheal node enlargement. Fifty percent of affected patients exhibit bilateral hilar adenopathy as the first manifestation of sarcoidosis. Regression of hilar nodes within one to three years occurs in 75 percent of such patients, while 10 percent develop chronic enlargement that can persist for ten years or more. Stage II consists of bilateral hilar adenopathy and reticular opacities. Reticular opacities occur predominantly in the upper lobe. Patients with stage II disease usually have mild to moderate symptoms, most commonly cough, dyspnea, fever, and/or easy fatigue. Stage III consists of reticular opacities with shrinking hilar nodes. Stage IV disease is characterized by reticular opacities with evidence of volume loss, predominantly distributed in the upper lung zones. Marked traction bronchiectasis, extensive calcification and cavitation, or cyst formation may be seen. From the above staging, this patient presented with Stage II sarcoidosis.

Chest CT can demonstrate a variety of abnormalities in patients with sarcoidosis [5, 6]. Hilar and mediastinal lymphadenopathy, beaded or irregular thickening of the bronchovascular bundles, nodules along bronchi, vessels and subpleural regions, bronchial wall thickening, ground glass opacification, parenchymal masses or consolidation, parenchymal bands, cysts, traction bronchiectasis, and fibrosis with distortion of the lung architecture.

A fluorine-18-fluorodeoxyglucose (18F-FDG) PET scan may be helpful to identify occult lesions and possibly reversible granulomatous disease [7]. This test does not differentiate sarcoidosis from malignancy, as 18F-FDG PET may be positive in both processes. However, in a small study (24 sarcoid, 17 lung cancer), the combination of 18F-FDG and 18F-FMT (L-[3-18F]-methyltyrosine) PET scanning was able to differentiate sarcoidosis from malignancy; sarcoid lesions were positive on 18F-FDG PET but negative on 18F-FMT PET (both are positive in patients with cancer) [8].

Sarcoidosis can involve all organ systems, which the most prominent sites of extrapulmonary disease include the skin, eyes, reticuloendothelial system, musculoskeletal system, exocrine glands, heart, kidney, and central nervous system. Extrapulmonary manifestations vary on the basis of sex, age at presentation, and ethnicity. Women are more likely to have neurologic or ocular involvement, while men more frequently have abnormalities of calcium homeostasis. Up to 30 percent of patients present with extrapulmonary disease [1].

In this patient, the maculopapular rash over the lower limb might represent the skin involvement, in which skin biopsy showed features suggestive of resolving vasculitis. Cutaneous involvement is seen in up to 20 percent of patients with sarcoidosis [9]. A maculopapular eruption is the most common subacute lesion. It usually involves the alae nares, lips, eyelids, forehead, rear of neck at the hairline, and/or previous trauma sites (for example, scars and tattoos). Waxy, pink nodular lesions are frequently distributed on the face, trunk, and extensor surface of the arms and legs. Plaque-like lesions can occur in chronic sarcoidosis including lupus pernio, a violaceous discoloration of the nose, cheeks, chin, and ears. Erythema nodosum (EN) is a component of Lofgren's syndrome and associated with a good prognosis and spontaneous remission [10]. Atyical lesions may be ulcerative, psoriasiform, hypopigmented, follicular, angiolupoid, rosacea-like, or morpheaform [11].

This patient has bilateral parotid swelling, however there was no uveitis or facial palsy to suggest Heerfordt's syndrome. Painless swelling of the salivary glands occurs in approximately 4 percent of patients with sarcoidosis. Xerostomia and keratoconjunctivitis sicca also may be seen, producing manifestations similar to those seen in Sjogren's syndrome. This patient complained of dry eyes and mouth however ophthalmologist assessment showed no evidence of keratoconjunctivitis sicca or anterior and posterior uveitis.

Reticuloendothelial system disease is common in sarcoidosis, manifest as peripheral lymphadenopathy (40 percent), hepatomegaly (20 percent), non-caseating granulomas on liver biopsy with or without hepatomegaly (75 percent), and splenic enlargement (25 percent). Hypersplenism can lead to anemia, leukopenia, and thrombocytopenia.

Calcium metabolism abnormalities are the most common renal and electrolyte abnormalities observed among patients with sarcoidosis. The defect in calcium metabolism is due to extrarenal production of calcitriol by activated macrophages. Patients may present with hypercalciuria (occurs in up to 50 percent of cases), hypercalcemia (which occurs in 10 to 20 percent), and nephrocalcinosis. If untreated, renal calcium deposition can lead to chronic renal failure and end-stage renal disease. The calcium level was normal in this patient however urinary calcium was not sent.

This patient had renal impairment, and CT showed bilateral enlarged kidneys with renal cysts, which might relate to sarcoid. Granulomatous infiltration of the kidney is not uncommon. Other renal complications of sarcoidosis include membranous nephropathy, a proliferative or crescentic glomerulonephritis, focal glomerulosclerosis, polyuria (due to nephrogenic and/or central diabetes insipidus), hypertension, and a variety of tubular defects. Computed tomography urography and renal biopsy will be helpful to determine the granuloma and exclude renal calculi.

Another finding in the CT scan was hepatomegaly of 22.4 cm in size with elevated serum alkaline phosphatase. These might be suggestive of granulomatous infiltration in the liver.

This patient had borderline leukocytosis and eosinophilia. Eosinophilia occurs in approximately 25 percent of patient. Anemia is uncommon, which results from the anemia of chronic disease. Hypersplenism and autoimmune hemolytic anemia can occur in some patients.

The erythrocyte sedimentation rate (ESR) is frequently elevated and a positive rheumatoid factor can exist. This coincides with the patient's results however the serum angiotensin converting enzyme (ACE) level was normal which against the diagnosis. ACE level is elevated in 75 percent of untreated patients with sarcoidosis [12]. False positive results occur in less than 5% of cases.

Bronchoalveolar lavage (BAL) can be used as an adjunctive measure to support the diagnosis of sarcoidosis by demonstrating a reduced number of CD8 cells, an elevated CD4 to CD8 ratio, and an increased amount of activated T cells, CD4 cells, immunoglobulins, and IgG-secreting cells [13, 14]. BAL can also be helpful in excluding infections as an alternative diagnosis. A CD4 to CD8 ratio less than one had a 100 percent negative predictive value for sarcoidosis. The triad of a CD4 to CD8 ratio greater than four to one, a lymphocyte percentage greater than or equal to 16 percent, and a transbronchial biopsy demonstrating non-caseating granulomas were the most specific tests for sarcoidosis. This combination of findings was associated with a 100 percent positive predictive value (PPV) for distinguishing sarcoidosis from other interstitial lung diseases, and an 81 percent PPV for distinguishing sarcoidosis from all other diseases.

D-dimer in BAL fluid also supports the diagnosis of sarcoidosis. One observational study found that eight out of ten patients with sarcoidosis had detectable D-dimer in their BAL fluid, compared to none of 18 healthy controls [15].

The characteristic morphologic feature of sarcoidosis is the non-caseating granuloma of the lung, which is most commonly found in the alveolar septa, the walls of bronchi, and the pulmonary arteries and veins. Granuloma formation is probably preceded by an alveolitis that involves the interstitium more than the alveolar spaces and is characterized by the accumulation of inflammatory cells, including monocytes, macrophages and lymphocytes [16].

The granuloma is a focal, chronic inflammatory reaction consists of epithelial cells, monocytes, lymphocytes, macrophages, and fibroblasts. Multinucleated giant cells are frequently found among the epithelioid cells within the granuloma follicle and often have cytoplasmic inclusions, such as asteroid bodies, Schaumann bodies, and birefringent crystalline particles (calcium oxalate and other calcium salts) [17]. Most sarcoid granulomas gradually resolve and leave few or no residual manifestations of previous inflammation.

The diagnosis of sarcoidosis requires compatible clinical and radiographic manifestations, exclusion of other diseases that may present similarly and histopathologic detection of non-caseating granulomas. Biopsies should be performed on accessible lesions, example from palpable lymph nodes, subcutaneous nodules, cutaneous lesions, an enlarged parotid gland, or an enlarged lacrimal gland. Erythema nodosum lesions should not be biopsied, because the histopathology will demonstrate panniculitis and not granulomas, even if sarcoidosis exists. Fiberoptic bronchoscopy with transbronchial biopsy should be performed if an accessible peripheral lesion cannot be identified. Open lung biopsy, thoracoscopic lung biopsy, or surgical mediastinal lymph node biopsy are options if bronchoscopy cannot be performed or is non-diagnostic.

In conclusion, this patient was referred to the rheumatology team for suspected Sjogren's syndrome. The clinical presentation was vague and atypical for sarcoidosis. Excision biopsy from the left cervical lymphnode showed non-caseating chronic granulomatous lymphadenitis, consistent with sarcoidosis.
